# Effect of Pillbox Organizers with Alarms on Adherence to Pharmacotherapy in Parkinson Disease Patients Taking Three and More Daily Doses of Dopaminergic Medications

**DOI:** 10.3390/jpm12020179

**Published:** 2022-01-28

**Authors:** Igor Straka, Michal Minar, Milan Grofik, Matej Skorvanek, Veronika Bolekova, Andrea Gazova, Jan Kyselovic, Peter Valkovic

**Affiliations:** 1 2nd Department of Neurology, Faculty of Medicine, Comenius University and University Hospital in Bratislava, 833 05 Bratislava, Slovakia; straka0105@gmail.com (I.S.); mmminar@gmail.com (M.M.); veronika.bolekova@gmail.com (V.B.); 2 Department of Neurology, Jessenius Faculty of Medicine, Comenius University and University Hospital in Martin, 036 01 Martin, Slovakia; milangrofik@gmail.com; 3 Department of Neurology, Faculty of Medicine, P.J. Safarik University and Pasteur University Hospital, 040 11 Košice, Slovakia; mskorvanek@gmail.com; 4 Faculty of Psychology, Institute of Clinical Psychology, Pan-European University, 821 02 Bratislava, Slovakia; 5 Faculty of Medicine, Institute of Pharmacology and Clinical Pharmacology, Comenius University, 811 08 Bratislava, Slovakia; aandreagazova@gmail.com; 6 5th Department of Internal Medicine, Faculty of Medicine, Comenius University and University Hospital in Bratislava, 826 06 Bratislava, Slovakia; kyselovic@gmail.com; 7 Department of Pharmacology and Toxicology, University of Veterinary Medicine and Pharmacy, 041 81 Košice, Slovakia; 8 Centre of Experimental Medicine, Institute of Normal and Pathological Physiology, Slovak Academy of Sciences, 813 71 Bratislava, Slovakia

**Keywords:** Parkinson’s disease, adherence, pharmacotherapy, quality of life, fluctuations, non-motor symptoms, pillbox organizer with alarm

## Abstract

Improvement of adherence to pharmacotherapy in patients with Parkinson’s disease (PD) is a challenge in routine clinical practice. Our study was aimed at the effect of pillbox organizers with alarms improving adherence to pharmacotherapy and its impact on clinical outcomes. Forty nonadherent patients with PD being treated with ≥ 3 daily doses of levodopa and/or dopamine agonists were pseudorandomized and consecutively ranked to groups A (early-start intervention) and B (delayed-start intervention). We used the following validated diagnostic instruments: MMAS-8 (adherence), PDQ-8 (quality of life, QoL), GDS (depression), NMSS (non-motor symptoms), MDS-UPDRS III (motor involvement), MDS-UPDRS IV, and WOQ-9 (motor and non-motor fluctuations and dyskinesias). We proved a significantly improved rate of adherence with the use of pillbox organizers with alarms. Moreover, after only four weeks of using the pillbox organizer, we detected an improvement in QoL scores, motor involvement, motor-, and non-motor fluctuations. Our study showed that pillbox organizers with alarms are efficient in improving adherence to pharmacotherapy in PD. It also could contribute to better motor states, less severe fluctuations, and improved QoL.

## 1. Introduction

Parkinson’s disease (PD) is a progressive neurodegenerative disorder with various motor and non-motor symptoms (NMS) that can potentially lead to a deterioration in the quality of life (QoL). Current options of the treatment are symptomatic. Dopaminergic therapy (levodopa and dopamine agonists) remains the golden standard to improve motor and non-motor symptoms. Short biological half-life of levodopa leads to pulsatile dopaminergic stimulation (compared to physiological continuous stimulation), which is considered to be the main cause of fluctuations and dyskinesias onset. Therefore, regular and timely-correct use of medication is crucial for more stable levodopa concentration and lower risk of complications [1]. Progression of PD requires increase in number of daily doses of medication what might impair adherence, increase disability, and further reduce QoL.

Adherence to therapy means the extent to which the patient’s behavior agrees with medical instructions of the physician [2]. It depends on several socioeconomic, therapy-related, patient-related, and health care team-related factors [3]. Non-adherence to pharmacotherapy in chronic disorders is an important healthcare problem connected with increased morbidity, mortality, and socioeconomic burden [4,5,6]. In patients with PD, suboptimal adherence varies between 10% and 67% [7] and is associated with motor and non-motor complications and reduced QoL [8,9,10,11].

To improve the rate of adherence, adequate communication with patients and their caregivers is essential. The use of dosing devices (such as pillbox organizers with alarms) did not yield consistent results in previous research in chronic disorders [12,13,14]. However, they may have the potential to improve adherence to pharmacotherapy in patients with PD [15]. Evidence about the effectiveness of pillbox organizers with alarms in the population affected by PD is limited.

The aim of this study was to find out (I) whether pillbox organizers with alarms would improve the rate of adherence in patients with PD taking three and more daily doses of PD drugs, and (II) how the improvement of adherence to pharmacotherapy affects the clinical outcomes.

## 2. Material and Methods

### 2.1. Participants

We enrolled patients who met the following criteria:

- idiopathic PD diagnosed according to the Movement Disorder Society (MDS) Clinical Diagnostic Criteria for PD [16],
- on standard dopaminergic medication with levodopa (plus dopa-decarboxylase inhibitor) and/or dopamine agonists in a minimum of three daily doses (therapeutic regimen had to be stable for a minimum of four weeks prior enrollment),
- without cognitive impairment (>26/30 points on the Mini-Mental State Examination, MMSE), and
- non-adherent patients who scored <6/8 points on the Morisky 8-Item Medication Adherence Scale (MMAS-8) [17].

Participants were recruited from movement disorder outpatient centers of University Hospitals in Bratislava, Košice, and Martin (Slovakia). Patients had to declare their ability to take antiparkinsonian medication independently. Eligible subjects could have a maximum of three controlled comorbidities. We excluded patients with deep brain stimulation and patients with pump therapies for the indication of PD.

Study procedures were performed according to the declaration of Helsinki, ethical aspects were approved by the local ethical committee for all centers, and all subjects signed written informed consent prior to inclusion into the study.

### 2.2. Methods

In our study, we used the following battery of tests and scales:

- the Morisky 8-Item Medication Adherence Scale (MMAS-8), to detect medication adherence [17],
- the 8-Item Parkinson’s Disease Questionnaire (PDQ-8) [18], to detect QoL,
- the Geriatric Depression Scale (GDS) [19,20], to detect depression,
- the Non-Motor Symptom Assessment Scale for Parkinson’s Disease (NMSS) [21], to detect frequency and severity of NMS,
- the Movement Disorder Society—Unified Parkinson’s Disease Rating Scale (MDS-UPDRS)—part III: Motor examination (MDS-UPDRS III), to detect motor score,
- MDS-UPDRS—part IV: Motor complications (MDS-UPDRS IV) [22], to detect motor complications, and
- the Nine-Item Wearing-off Questionnaire (WOQ-9) [23], to detect wearing-off phenomenon (defined as motor and NMS fluctuations).

In all scales and questionnaires (excluding MMAS-8), higher scores were associated with more severe symptoms.

Patients completed the MMAS-8, PDQ-8, and WOQ-9. Trained investigators administered the MMSE, GDS, NMSS, MDS-UPDRS III, and IV. Baseline characteristics (age, disease duration, previous medication history, comorbidities) and modified Hoehn & Yahr score (H&Y) [24] were recorded by patients and investigators.

### 2.3. Pillbox Organizer with Alarm

**Figure 1:**
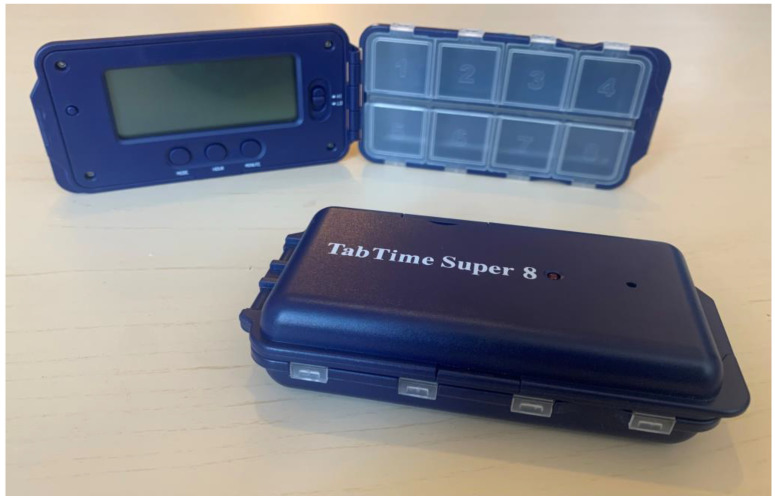
TABTIME^®^ Super 8.

For the purposes of this study, we used pillbox organizers with alarms—TABTIME^®^ Super 8 (TabTime Ltd, Moss Ln, Sandbach, UK) (size 12 × 6 × 3 cm, Figure 1). This tool has eight tablet compartments and up to eight daily alarms (audio and visual) set to specifically required times. An audio alarm rings for 30 s, and a visual LED reminder light continues to flash until the pillbox is opened. All participants of the study were trained in the use of the pillbox organizers with alarms, and times of doses were set by investigators.

### 2.4. Study Protocol

Subjects with low levels of adherence (<6/8 points on MMAS-8) were pseudorandomized and consecutively ranked to groups A and B. Odds were assigned to group A, and evens to group B.

Group A (early-start intervention) consisted of subjects who received pillbox organizers with alarms (and were instructed on their use) after passing the set of tests on a baseline (“null”) visit (V0). The first follow-up visit (V1) during which subjects passed the same battery of tests and questionnaires, except for MMSE, was four weeks after the baseline visit. Patients could voluntarily continue to use the organizers (without further instructions). Four weeks later, the patients were invited for a second follow-up visit (V2) and passed the same set of assessment tests as in V1.

Group B (delayed-start intervention) consisted of subjects who did not receive any tool for adherence improvement (neither pillbox organizers with alarms, nor specific instructions regarding adherence). At the baseline (“null”) visit (V0), they completed the same examinations as Group A. Four weeks later, they completed a follow-up visit (V1) with the same protocol as Group A. This period served as the control to group A. Only after completing this visit (V1) did Group B subjects receive pillbox organizers with alarms (and were instructed on their use). After four weeks, they completed the second visit (V2), as did Group A.

**Figure 2:**
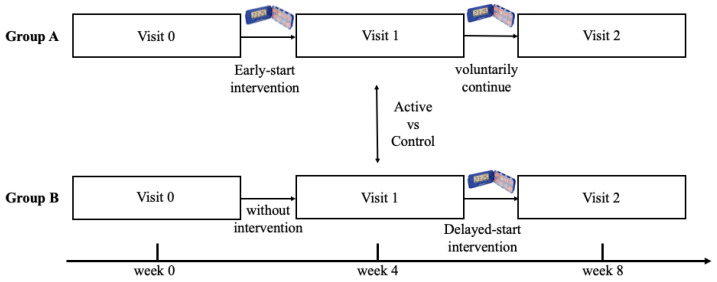
Graphical presentation of the protocol.

Graphical presentation of the protocol is shown in Figure 2. The overall therapeutic regimen must remain unchanged during the whole duration of the study.

### 2.5. Statistical Analysis

Statistical analysis of data was performed using IBM^®^ SPSS^®^ Statistics for Windows, Version 26.0 (IBM Corp., Armonk, NY, USA). To analyze basic demographic and clinical parameters, we used descriptive statistics. We used a nonparametric Wilcoxon test for paired values (for testing time effect of intervention) of the statistical hypothesis of median equality in visits. For testing intergroup differences, we used a nonparametric Mann–Whitney test (effect of intervention is in V1). To counteract mistakes in multiple comparisons, we used Bonferroni correction. *p*-value ≤ 0.05 was considered to be significant. Effect size measures were assessed by *r* (small effect < 0.299; moderate effect 0.300–0.449; and large effect ≥ 0.500).

## 3. Results

We included 40 subjects with idiopathic PD. Demographic and basic clinical data are presented in Table 1.

Table 2 shows the results of a pairwise comparison of Group A (early-start intervention) and Group B (delayed-start intervention) at baseline visit (V0) and visits after four (V1) and eight weeks (V2). In Group A (early-start intervention), we found increased rates of adherence (MMAS-8: *p* < 0.001; *r* = 0.891), as well as improvements in the score of quality of life (PDQ-8: *p* < 0.001; *r* = 0.814), in the evaluation of motor scores (MDS-UPDRS III: *p* < 0.01; *r* = 0.671), and in the scores of motor and non-motor complications (MDS-UPDRS IV: *p* < 0.01, *r* = 0.718; WOQ-9: *p* < 0.01, *r* = 0.770), after four weeks of using the pillbox organizers with alarms (V0 versus V1). Comparing V1 and V2, we found worsened parameters of QoL, as well as motor scores, and motor and non-motor complications (12 out of 20 participants volunteered to continue using the pillbox organizer after V1). However, comparing V0 and V2, the effect of the intervention was still large in all mentioned parameters (*r* value ranging from 0.534 to 0.887 for various parameters).

In Group B (delayed-start intervention), the effect size of differences between V0 and V1 were small. After intervention (V1 versus V2), we found improvement in the same parameters as Group A (*r* value ranging from 0.668 to 0.889).

By comparative analysis of the groups A and B at time of V1 (Table 3), we found significant differences only in the rate of adherence (MMAS-8: *p* < 0.001, *r* = 0.811).

## 4. Discussion

Our results show that patients who used the pillbox organizers with alarms for four weeks had significantly better adherence compared to patients without this intervention.

We did not find any equivalent or similar research of this subpopulation in literature. The effect of alarmed pillbox organizers was not unambiguously confirmed in other chronic disorders, such as arterial hypertension or diabetes [25,26,27]. However, in comparison with these conditions, any missed dose of antiparkinsonian medication, especially in more advanced diseases, can have a direct and rapid effect on the deterioration of motor and non-motor state of patients with PD [11]. Nevertheless, we detected improvements in motor scores after 4 weeks of using pillbox organizers with alarms; lower adherence rates have been already confirmed to worsen motor state in PD [7,10,11,28,29,30,31,32,33]. Group A (early-start), which was allowed to continue using pillbox organizers for the following four weeks (without further instructions, imitating more natural conditions, or real-life practice), reported significantly better adherence at the end of the second follow-up compared to baseline.

Long-term treatment with relatively high doses of levodopa leads to motor fluctuations and dyskinesias in most patients with PD [34]. It is caused by pulsatile (not continuous or irregular) dopaminergic stimulation. Dopamine agonists with prolonged or sustained release delayed onset of treatment complications by three years, which is closer to the concept of continuous dopaminergic stimulation [35,36]. As previous research shows, non-adherence is strongly associated with more severe motor complications (fluctuations and dyskinesias) [10,28,37,38]. Our study indicated that fluctuations were reduced (according to MDS-UPDRS IV and WOQ-9 score) after 4 weeks of using the pillbox organizer with alarm in both groups. In our opinion, it may be caused by improving overall adherence, as well as time adherence. However, the intergroup difference between groups A and B at time of V1 was not significant, which can be caused by placebo effect of short-time intervention. However, further studies are needed to verify the effectiveness of this method.

Contrarily, we did not detect any reduction in the frequency and severity of NMS, which can be explained by the fact that some NMS are caused by the disruption in non-dopaminergic systems [39]. Moreover, NMS, such as excessive daytime sleepiness, anhedonia, anxiety, forgetfulness, and falls, predict lower levels of adherence to PD medication [10,33,38]. We did not identify improvement in the score of depression (GDS), despite the improvement of adherence to dopaminergic PD medication with subsequent reduction of motor fluctuations. While depression may be present as part of non-motor fluctuations resulting from fluctuating dopaminergic levels, the involvement of non-dopaminergic neurotransmitters in the pathophysiology of depression may explain this result. Moreover, depressive symptomatology is crucial in the contribution of decreased adherence to medication [40].

Several researchers recently referred to the fact that adherent patients with PD have better QoL [10,30,41]. After four weeks of observation, we detected improvement of QoL.

In our study, we identified intergroup differences in the first follow-up visit (after 4 weeks in active Group A and control Group B) only in the scores of adherence. Despite a trend to improved scores in other parameters, we did not find further significant differences in our findings. This could have been influenced by some limitations, such as a low number of participants and a relatively short study duration. As we already mentioned, long-term adherence to pillbox organizers in chronic diseases is not sufficient (e.g., arterial hypertension, diabetes) [25,26,27] but, in the case of PD, any irregularity and non-adherence to pharmacotherapy might lead to immediate worsening of clinical state. Another limitation of our study was the usage of subjective questionnaires, and the fact that raters were not blinded. However, we did not consider having blinded raters to be crucial as the majority of the scales were self-administered or based on subjective assessment. In addition, the willingness of subjects to participate in the study may have partially led to improved adherence through increased motivation. Therefore, further studies with more subjects and longer duration are needed.

In summary, complex therapeutic regimens are associated with lower adherence, a serious treatment-related problem, associated with worse medical outcomes, deteriorated QoL, increased morbidity and mortality, and higher economic burdens. Therefore, active screening of non-adherence in routine clinical practice is crucial. It is also necessary to develop methods and/or devices for adherence improvement. Our study indicates that pillbox organizers with alarms are a potentially efficient intervention in patients with PD who take three or more daily doses of antiparkinsonian drugs, by improving time and total adherence.

## Figures and Tables

**Table 1:** Descriptive statistics of basic demographic and clinical data.

Demographic and Clinical Data	Together	Group A (*n* = 20)	Group B (*n* = 20)	*p*
Men	26 (65.00%)	12 (60.00%)	14 (70.00%)	0.507
Women	14 (35.00%)	8 (40.00%)	6 (30.00%)	
Age (year)	68.50 (13.50)	68.00 (8.75)	69.50 (16.50)	0.659
Duration of PD (year)	7.00 (4.00)	7.50 (3.75)	7.00 (3.75)	0.096
H&Y	2.5 (1.00)	2.5 (0.88)	2.5 (1.00)	0.371
LEDD	1314.00 (611.00)	1185.00 (638.50)	1367.50 (628.13)	0.799
Number of PD drug/day	7.00 (4.75)	9.00 (6.00)	7.00 (2.75)	0.253
Number of PD doses/day	5.00 (1.75)	5.00 (2.00)	5.00 (1.00)	0.841
Fluctuating patients (according to MDS-UPDRS IV)	27 (67.50%)	15 (75.00%)	16 (80.00%)	0.705

Numerical variables presented as median (interquartile range); categorical variables (gender, number of fluctuating patients) presented as number (%). H&Y—Hoehn & Yahr score, LEDD—levodopa equivalent daily dose (mg), MDS-UPDRS IV—MDS-Unified Parkinson’s Disease Rating Scale—part IV: Motor complications, PD—Parkinson’s disease.

**Table 2:** Effect of intervention with pillbox organizer with alarm (Wilcoxon test).

Scales and Questionnaires		V0	V1	V2	Wilcoxon Test								
		Median (IQR)	Median (IQR)	Median (IQR)	V0–V1			V1–V2			V0–V2		
					Z	*p*	*r*	Z	*p*	*r*	Z	*p*	*r*
MMAS-8	A	4.00 (2.00)	7.00 (1.00)	6.00 (1.00)	−3.985	<0.001	0.891	3.333	0.999	0.745	−3.967	**<0.001**	0.887
	B	4.50 (1.00)	4.00 (1.00)	7.00 (0.75)	−1.342	0.540	0.300	−3.975	**<0.001**	0.889	−3.975	**<0.001**	0.889
PDQ-8	A	8.50 (10.00)	7.00 (7.50)	7.50 (6.75)	−3.639	**<0.001**	0.814	−0.162	0.999	0.036	−3.401	**0.003**	0.760
	B	9.50 (9.00)	10.00 (9.50)	7.00 (7.50)	−1.732	0.249	0.387	−3.535	**<0.001**	0.790	−3.531	**<0.001**	0.790
GDS	A	9.00 (9.50)	9.50 (7.50)	9.00 (7.00)	−1.103	0.810	0.247	−1.107	0.804	0.248	−0.082	0.999	0.018
	B	10.00 (10.50)	10.00 (9.75)	11.00 (12.25)	−0.500	0.999	0.112	−1.637	0.306	0.366	−1.457	0.435	0.326
NMSS	A	64.50 (49.00)	65.50 (44.25)	64.00 (45.75)	−1.645	0.300	0.368	−0.044	0.999	0.010	−1.572	0.348	0.352
	B	76.50 (50.75)	76.00 (51.00)	75.50 (49.50)	−0.526	0.999	0.118	−1.164	0.735	0.260	−1.727	0.252	0.386
MDS-UPDRS III	A	33.00 (10.75)	31.00 (10.75)	31.00 (11.50)	−2.999	**0.009**	0.671	−1.811	0.210	0.405	−2.389	0.051	0.534
	B	35.00 (13.50)	35.00 (13.75)	32.00 (12.50)	−0.957	0.999	0.214	−3.275	**0.003**	0.732	−2.441	**0.045**	0.546
MDS-UPDRS IV	A	7.00 (9.25)	5.00 (7.00)	5.00 (6.75)	−3.209	**0.003**	0.718	−0.557	0.999	0.125	−3.238	**0.003**	0.724
	B	7.00 (6.50)	7.00 (6.00)	4.50 (4.00)	−1.633	0.306	0.365	−3.438	**0.003**	0.769	−3.184	**0.003**	0.712
WOQ-9	A	4.00 (3.00)	2.00 (3.00)	2.00 (2.00)	−3.442	**0.003**	0.770	−1.265	0.618	0.283	−3.602	**<0.001**	0.805
	B	4.00 (2.75)	3.50 (3.00)	2.00 (1.00)	−1.414	0.471	0.316	−2.986	**0.009**	0.668	−3.114	**0.006**	0.696

Numerical variables presented as median (IQR—interquartile range). V0—baseline visit, V1—first follow-up visit, V2—second follow-up visit, A—group A, B—group B, GDS—Geriatric Depression Scale, MMAS-8—8-Item Morisky Medication Adherence Scale, MDS-UPDRS III—MDS-Unified Parkinson’s Disease Rating Scale—part III: Motor examination, MDS-UPDRS IV—MDS-Unified Parkinson’s Disease Rating Scale—part IV: Motor complications, NMSS—Non-Motor Symptom Assessment Scale for Parkinson’s disease; PDQ-8—8-Item Parkinson’s Disease Questionnaire, WOQ-9—9-Item Wearing-off Questionnaire. Bold is used to highlight statistical significance, *p* ≤ 0.05.

**Table 3:** Differences in the level of assessed variables between groups A (early-start intervention) and (A) and B (delayed-start intervention) (Mann–Whitney test). Timepoint V0 represents difference between groups A and B in baseline. Timepoint V1 represents difference between active and control group.

Scales and Questionnaires	Visit	Mann-Whitney U	Z	*p*	*r*
MMAS-8	V0	154.50	−1.311	0.663	0.207
	V1	16.00	−5.127	**<0.001**	0.811
	V2	123.50	−2.315	0.114	0.366
PDQ-8	V0	199.00	−0.027	0.999	0.004
	V1	137.00	−1.710	0.273	0.270
	V2	191.00	−0.245	0.999	0.039
GDS	V0	166.50	−0.909	0.999	0.144
	V1	175.50	−0.664	0.999	0.105
	V2	184.00	−0.434	0.999	0.069
NMSS	V0	190.00	−0.271	0.999	0.043
	V1	189.00	−0.298	0.999	0.047
	V2	185.00	−0.406	0.999	0.064
MDS-UPDRS III	V0	164.00	−0.975	0.999	0.154
	V1	134.50	−1.774	0.228	0.280
	V2	173.00	−0.731	0.999	0.116
MDS-UPDRS IV	V0	197.00	−0.082	0.999	0.013
	V1	137.50	−1.707	0.273	0.270
	V2	193.00	−0.191	0.999	0.030
WOQ-9	V0	191.00	−0.247	0.999	0.039
	V1	136.00	−1.754	0.258	0.277
	V2	173.00	−0.748	0.999	0.118

V0—baseline visit, V1—first follow-up visit, V2—second follow-up visit, GDS—Geriatric Depression Scale, MMAS-8—8-Item Morisky Medication Adherence Scale, MDS-UPDRS III—MDS-Unified Parkinson’s Disease Rating Scale—part III: Motor examination, MDS-UPDRS IV—MDS-Unified Parkinson’s Disease Rating Scale—part IV: Motor complications, NMSS—Non-Motor Symptom Assessment Scale for Parkinson’s disease; PDQ-8—8-Item Parkinson’s Disease Questionnaire, WOQ-9—9-Item Wearing-off Questionnaire. Bold is used to highlight statistical significance, *p* ≤ 0.05.

## Data Availability

The data presented in this study is available upon request from the corresponding author. The data are not publicly available because the database contains patient personal data.
